# Critical Overview on Pure Chitosan-based Scaffolds for Bone Tissue Engineering: Clinical insights in Dentistry

**DOI:** 10.7150/ijms.87978

**Published:** 2023-09-18

**Authors:** Luca Signorini, Gaetano Marenzi, Anastasia Facente, Benedetta Marrelli, Rosa Maria Marano, Alessandra Valletta, Luciano Pacifici, Roberta Gasparro, Gilberto Sammartino, Marco Severino

**Affiliations:** 1 Saint Camillus University of Health Science, 00100 Rome, Italy.; 2 Department of Neurosciences, Reproductive and Odontostomatological Sciences, Postgraduate School of Oral Surgery, University “Federico II” of Naples, via S. Pansini 5, 80131 Naples, Italy.; 3 Tecnologica Research Institute - Marrelli Health, 88900 Crotone, Italy.; 4 Department of Oral and Maxillo-Facial Sciences, Sapienza University of Rome, 00195 Rome, Italy.; 5 Department of Medicine and Surgery, University of Perugia, Italy.

## Abstract

Bone Tissue Engineering (BTE) is a field of regenerative medicine continuously improving, thanks to the development of new biomaterials used as grafts or scaffolds for repairing bone defects. In recent years, chitosan, a natural biopolymer extracted mainly from crustacean shells, has demonstrated unique and desirable characteristics for BTE applications, such as: biocompatibility, biodegradability, and osteoconductive behavior. Additionally, the presence of numerous active amine groups in its chemical structure allows it to be easily modified. Data suggest that chitosan scaffolds are highly biomimetic, and show an interesting bioactivity, and antibacterial behavior. We have demonstrated, in a critical overview, how chitosan-based scaffolds may hold great interest for BTE applications in medical and dental applications. Future research should be focused on the use of chitosan-scaffolds combined with other biomaterials or bioactive molecules, to increase their overall regenerative potential, also in critical-sized defects. In conclusion, chitosan can be considered a promising biomaterial in BTE and clinical dentistry.

## 1. Introduction

In recent years, novel and bioactive biomaterials have been extensively studied for biomedical applications. In particular, scaffolds fabricated with innovative biomaterials have been investigated to overcome the limitations in the healing of critical sized bone defect. An important aspect of Bone Tissue Engineering (BTE) is the fabrication of implantable scaffolds, which contribute to bone regeneration by providing a suitable environment for osteogenic stem cells to differentiate and regenerate new bone.

**Figure 1:**
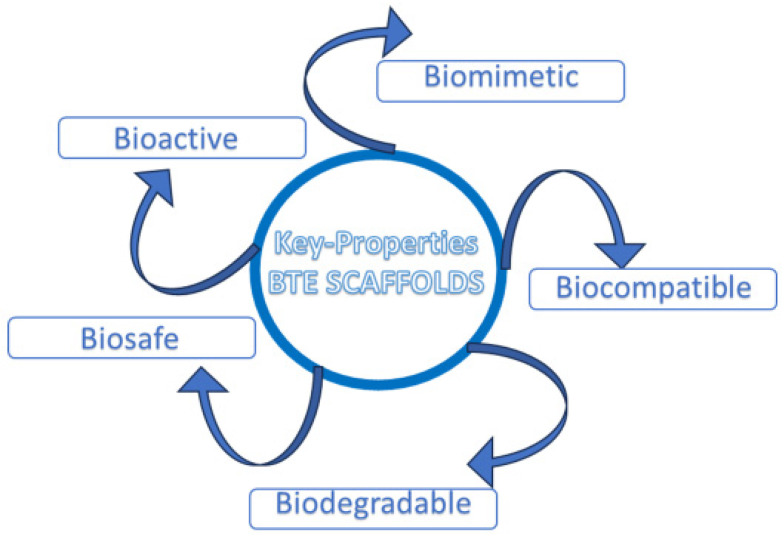
Key properties of ideal BTE scaffold.

Therefore, an ideal scaffold for BTE should satisfy specific requirements, such as: biocompatibility, tunable biodegradability, good porosity to ensure an efficient diffusion of nutrients, and a proper neo-angiogenesis; scaffolds should also ensure adequate mechanical properties, including a homogeneous cell-to-biomaterial attachment 1 **(Fig. 1)**.

Bone defects resulting from trauma, degenerative conditions, surgery, or congenital malformations, are a significant health issue; in fact, bone tissue is the second most transplanted tissue just after blood 2. Treatment of broken or damaged bone can be carried out using a biodegradable compound, employed as a temporary skeleton to replace lost bone or defective sites. The designed and fabricated bone support should be gradually degraded and replaced by the new bone tissue, with no adverse effect on the health of the body 3.

In this context, researchers have focused their attention on the possible use of polymeric materials, i.e., organic materials (of natural or synthetic origin), composed of long chains of atoms, joined by covalent bonds. The ideal polymer for tissue engineering should be: (1) mechanically stable, (2) biocompatible and bioactive, and (3) biodegradable in a short (one month for soft tissue) or a long time (from six month to one year for hard tissue like bone) 4,5.

In view of the chemical-structural composition of natural bone, which consists of organic substances (collagen and non-collagenous proteins), inorganic substances (hydroxyapatite and impurities) and water, in which hydroxyapatite crystals are neatly distributed around collagen fibers 6, scientific evidence have suggested that natural polymers are able to mimic the extracellular matrix (ECM) and thus provide a favorable microenvironment for cell growth 7.

In the present work, attention has been focused on the use of chitosan as a polymer matrix for the fabrication of scaffold that exhibits physicochemical characteristics similar to natural bone and osteogenic properties that make it a promising material to be applied in the field of bone regenerative medicine and, thus, act as a porous microenvironment that promotes bone/odontogenic attachment, viability and differentiation.

## 2. Chitosan

Chitosan, a cationic linear polysaccharide obtained by deacetylation of chitin, is one of the biomaterials abundantly used in biomedical, biopharmaceutical, as well as food industry. It consists of repeated units of ꞵ-(1-4) N-acetylglucosamine and D-glucosamine, with active amine groups undergoing protonation at physiological pH (pH=7.4). It is a polymeric compound soluble in acidic aqueous solutions, such as acetic acid and lactic acid; however, it has poor solubility in neutral and basic environments. The solubility of chitosan depends on many factors, such as the degree of deacetylation, pH, molecular weight, temperature, and crystallinity of the polymer itself. It is widely documented in the literature that a high degree of deacetylation and a low molecular weight improve the degree of solubility of the material 8.

It exhibits remarkable properties such as: biodegradability, antibacterial activity, high bioavailability, biocompatibility, and good miscibility with other polymeric materials. In addition, it has no risk of toxicity, therefore it has been approved by the *Food and Drug Administration* (FDA) for biomedical and biopharmaceutical applications 9,10. Chitosan has been widely used in the regeneration of certain human tissues (bone, cartilage, cardiac tissue, corneal tissue, periodontal tissue), wound healing, gene delivery, and even cosmeceutical applications 11.

Based on research findings, it could mediate potential beneficial effects on the body's health; in fact, it has been shown to improve peristalsis in the small intestine, reduce blood pressure, lower blood cholesterol levels, and boost immune response 12.

Biodegradability is a crucial property in the development of new biopolymers. In the organisms, the biodegradability of chitosan depends on many factors, including the degree of deacetylation (DD) and molecular weight (MW). Degradation mechanisms affecting chitosan include physical processes (swelling, cracking, and dissolution) and chemical processes, namely depolymerization, oxidation and hydrolysis (enzymatic and nonenzymatic) 12. These processes can be simulated *in vitro* and observed *in vivo* 13. However, given the complexity of the biological/physiological environment of the organism, *in vitro* degradation studies can only estimate the behavior of the materials *in vivo*. Chitosan is subject to enzymatic hydrolysis by lysozyme, a proteolytic enzyme present in the tissues of all humans. Similarly, lipase (present in human gastric, pancreatic fluid or saliva) is capable of degrading chitosan. In all cases, the products resulting from its degradation do not exhibit toxicity. Enzymatic degradation by lysozyme can basically be schematized in 3 phases: first, in which there is rapid weight loss and water absorption; second (termed static), in which lysozyme and water permeate into the crystalline zone; and last, in which the crystalline zone is degraded by lysozyme 12,13.

Availability, easy processability, biological properties and low cost make it a versatile biopolymer for scaffold design and fabrication in BTE.

## 3. Chitosan scaffold

Previous *in vitro* studies found the chitosan scaffold promote cell adhesion and proliferation, neovascularization, mineralization, and osteogenic morphogenesis for bone rejuvenation 14. Additionally, it induces the growth of calcium phosphate crystals, a precursor of hydroxyapatite and led to increase the expression of alkaline phosphatase (ALP), a marker of early-stage osteogenesis 15.

Several conventional techniques have been proposed to fabricate porous chitosan structures, which include freeze-drying, gas foaming, solvent casting, particle leaching, electrospinning, and 3D printing/Bioprinting 16.

Each fabrication technique shows numerous advantages (pros) and disadvantages (cons), which should not be underestimated. However, the ideal fabrication technique has not yet been discovered; in fact, further studies are needed to investigate possible solutions to overcome the limitations that may arise. In addition, often, scaffolds consisting of only one component may not meet the requirements for complete repair of the damaged/injured tissue or defect. Therefore, the fabrication of multicomponent scaffolds (composite/hybrid scaffolds) may be useful to address the complex requirements of bone defect repair.

## 4. Chitosan modifications

Despite the many positive features, chitosan has also many limitations, drawbacks that can often limit its use. Recently, there has been a growing interest in structure modification of chitosan to improve the solubility of these compounds and widen their applications in tissue engineering. Therefore, modifying the structure of chitosan could be a strategy to be implemented to expand the range of its applications. Over time, many possible modifications have been experimented with to achieve the desired physicochemical characteristics: by physical, chemical, and enzymatic means 17-20. Usually, chemical, and physical reforms necessitate the use of reagents and the application of harsh conditions. Thus, with rising safety and environmental concerns, enzymatic modification of chitosan is anticipated to be a common approach for safe chitosan modification 21.

Several studies showed that CS with substituent incorporation can decrease intracellular reactive oxygen species (ROS), thereby boosting the osteogenic differentiation of mesenchymal stem cells (MSCs) 22.

The general approaches used to include *cross-linking*, structure modification, and *grafting* with biodegradable polymer 23. Depending on the nature of the polymer matrix and functional groups, chitosan could be cross-linked using various methods, including physical, chemical, and enzymatic approaches or a combination of these. The main purpose of cross-linking is to improve the biomechanical properties of the scaffolds through the formation of a solid network in the polymer matrix. In addition, it can also modify the antigenic sites of natural materials and reduce their antigenicity 24.

The graft copolymerization method is considered a promising approach for the development of highly sophisticated functions 25-28. *Grafting* parameters (*grafting* percentage and its efficiency) are strongly influenced by the initiator used, its concentration and type, as well as reaction time and temperature. Several studies have been conducted in order to investigate the possible influence these parameters may exert on the grafting percentage and the characteristics of the grafted material 29.

The main disadvantage that chitosan has shown is the lack of adequate mechanical strength. Therefore, several studies have been conducted involving the combination of chitosan with other materials (ceramic, polymeric, or metallic) 30.

In addition, synthetic polymeric materials that have found wide use for biomedical applications include Poly-Glycolic Acid (PGA), Poly-Lactic Acid (PLA), and Poly-Caprolactone Acid (PGCL) 31. Belonging to the class of aliphatic polyesters, they are particularly interesting because they are biocompatible, nontoxic, and their degradation products (lactic acid, glycolic acid) are easily disposed of by the host organism 32. It has been shown that the content of these synthetic materials affects the three-dimensional structure and porosity of composite scaffolds 33. In detail, the compressive strength of the composite material increases proportionally as the content of PGA, PLA, and PGCL increases. However, an excessive amount can lead to the decline of the mechanical properties of the materials themselves, making the structure more compact and accelerating its degradation which is not conducive to bone regeneration 34. The advantage of using these synthetic polymeric compounds is due to the possibility of programming the scaffold degradation time according to its application.

A number of researchers have shown that the incorporation of mesoporous zinc silicate (mZS), within the matrix of chitosan scaffolds, can improve some of their properties, such as porosity, degradation rate, and biomineralization 1.

In addition, several strategies have been proposed to modify chemically chitosan using bioactive molecules (e.g., polyphenolic compounds) to impart or enhance some key biological properties (antioxidant, antimicrobial, anti-inflammatory, antitumor) 23.

Although investigators have fabricated a variety of biomaterials that mimic the morphology and functions of natural bone tissue and have verified the effectiveness to promote bone regeneration, there are still many limitations and obstacles in the BTE application of modified chitosan 35,36.

## 5. Chitosan scaffold fabrication procedure

**Figure 2:**
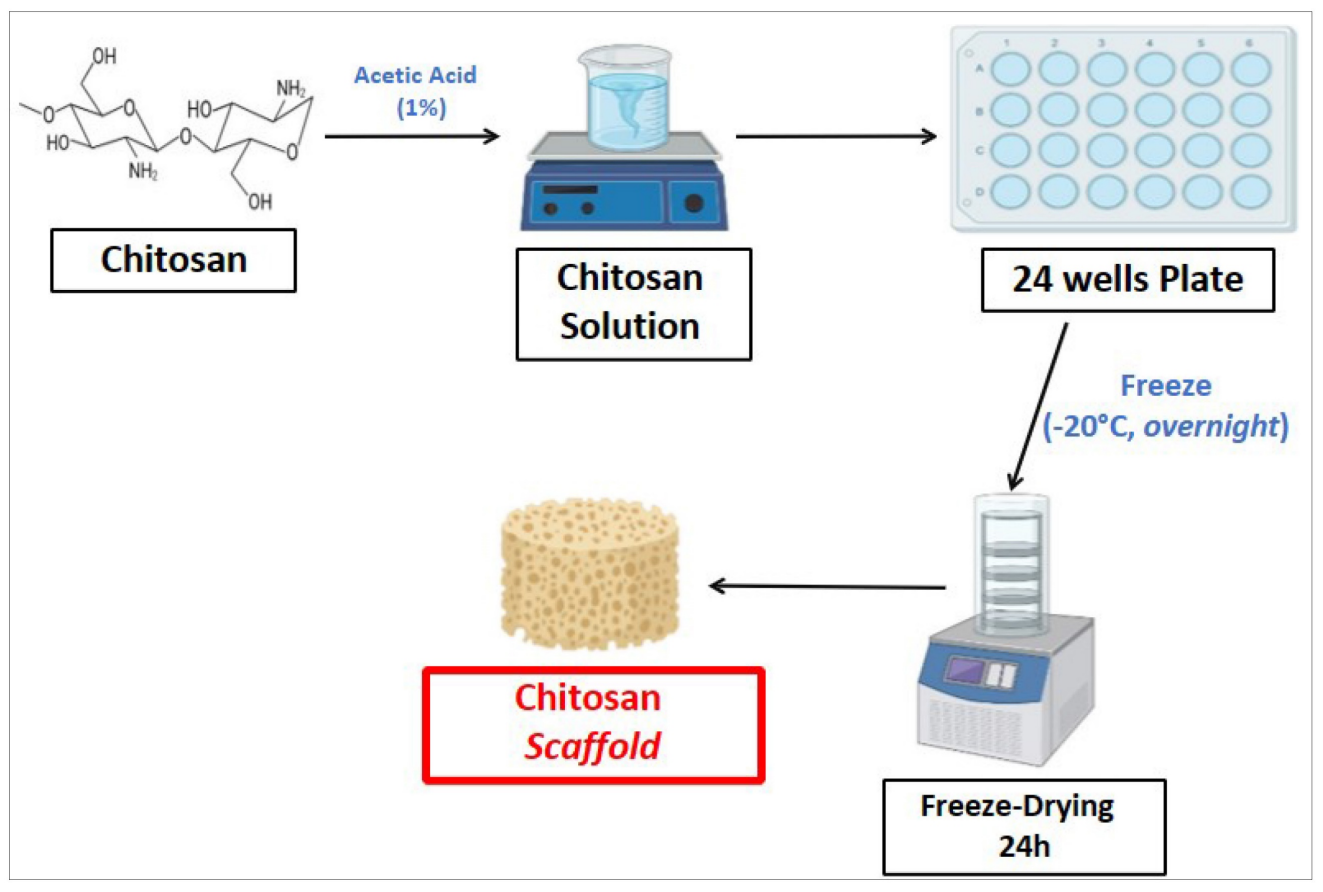
Chitosan scaffold fabrication procedure

Chitosan scaffold fabrication procedure was performed as follows. Briefly, chitosan was dissolved in an aqueous solution of acetic acid (1%) at room temperature for 24 hours, under constant stirring condition. The resulting chitosan solution was cooled to room temperature, poured into 24-well plates and placed in a freezer at -20°C overnight. Then, the frozen mixture was freeze-dried for 24 hours at -80°C in order to obtain chitosan scaffolds, with porous consistency. Figure 2 shows the procedure to fabricate chitosan scaffold.

## 6. Discussion

In recent years, different scaffolds have been proposed to the field of regenerative medicine and bone tissue engineering, to repair bone defects and its function 37-40. Despite of the achievements, further studies are still needed to provide a foundation for clinical applications/treatments 41,42.

The main factors that were taken into consideration to create chitosan scaffolds include: (1) Size and structure of the scaffold (larger scaffolds require more time for degradation); (2) Degree of deacetylation; (3) Molecular weight (as the molecular weight increases, the rate of swelling and degradation decreases); (4) Percentage of chitosan in the construct (higher chitosan content could increase the packing of the chains, increasing their crystallinity and decreasing the rate of degradation). *In vitro* characterization has been shown that chitosan scaffold exhibited physicochemical, mechanical and biocompatibility properties adequate for the purpose of the research.

Two main parameters were important to fabricate adequate scaffolds: density and porosity. Specifically, porous scaffold plays a key role in the process of tissue repair/regeneration as it preserves tissue volume, promotes cell migration, provides temporary mechanical structure, and ensures adequate supply of bioactive molecules. High density of the construct can lead to greater mechanical stability, and optimal porosity is an important permeability factor that promotes the distribution of nutrients through the scaffold and the removal of waste substances from it 43.

The degradation rate of chitosan is inversely related to the degree of crystallinity, which depends on the deacetylated forms; in particular, if highly deacetylated (DDN>85%) they have a relatively low degradation rate and can last for months* in vivo*, otherwise they are last less. Several studies have been documented that the degree of deacetylation is inversely related to the rate of degradation. In additional, the degradation rate greatly affects the mechanical and solubility properties 44,45. The degree of chitosan deacetylation also depends on the organism's response; in fact, a lower degree of deacetylation corresponds to less implant rejection 46, whereas a high degree of deacetylation corresponds to lower cell viability.

The many properties shown identify chitosan as a potential candidate for use in a wide variety of applications. On the other hand, chitosan exhibits similar structure to glycosaminoglycans (GAGs), a major component of the extracellular matrix (ECM) of tissues, which is why it is widely used in tissue regeneration and restoration, due in part to its biodegradability and cell affinity 3. The mimicry of osteogenic ECM is an important component for the creation of biomimetic scaffolds for application in BTE. By virtue of its structural similarity to the ECM of bone tissue and its inherent immune-stimulating properties (favorable to the local healing process), chitosan is an ideal candidate, offering significant advantages over alternatives, such as synthetic polymers, hybrids, or ceramic 47. There were sufficient findings to support osteogenic effect of chitosan in tissue engineering.

Preclinical and clinical studies have highlighted the important role mediated by mesenchymal stem cells (MSCs) in tissue homeostasis and their potential use in tissue engineering and the field of regenerative medicine. Their possible use in the treatment of a wide variety of pathologies has been suggested, as they can modulate inflammation, enhance angiogenesis, reduce tissue fibrosis, exhibit multipotential differentiation capacity and migratory properties 48, 49. In this context, DPSCs represent a very promising oral MSC source. In particular, these cells showed *in vitro* multilineage differentiation potential for osteo/odontogenic, adipogenic, chondrogenic, neurogenic, angiogenic and myogenic lineages 50, while *in vivo* studies have confirmed their enhanced potential to reconstitute mineralized tissues, including bone 51 and dentine/pulp complex 52. These results are consistent. It could be concluded that the above results confirmed that chitosan scaffolds promoted the osteogenic differentiation of DPSCs and pointed out the direction for further study of the mechanism.

Chitosan scaffold has similar physicochemical characteristic as natural bone also including commendable osteogenic properties that make it a promising material in the field of BTE. Data suggested that chitosan scaffold not only showed good biocompatibility, bioactivity, and antibacterial ability, but also significantly promoted osteoblastic differentiation and mineralization *in vitro*. Thus, the chitosan scaffold holds great promise for bone tissue engineering applications based on their robust mechanical properties, osteoconductive behavior, and antibacterial activities.

Analyzing the text in the context of dentistry and regenerative dentistry, the potential translational aspects of chitosan in these fields become evident. Chitosan's strategic properties, including biocompatibility, biodegradability, and osteoconductive behavior, make it an appealing candidate for various applications in dentistry 53, 54. For instance, in the field of dentistry, chitosan-based scaffolds could offer promising solutions for periodontal tissue engineering. Periodontal diseases, such as periodontitis, often lead to the loss of supporting tissues around teeth. Chitosan scaffolds, when combined with suitable bioactive molecules and growth factors, can provide a favorable microenvironment for periodontal tissue regeneration. By promoting cell adhesion, proliferation, and differentiation, chitosan scaffolds may facilitate the regeneration of damaged periodontal tissues and restore proper tooth support.

Furthermore, chitosan-based materials could find applications in dental implantology. Dental implants require optimal bone integration for long-term success. Chitosan scaffolds, in combination with other biomaterials, could enhance osseointegration by providing a supportive framework for bone growth around the implant site. By stimulating the recruitment and differentiation of osteogenic cells, chitosan scaffolds may contribute to improved implant stability and long-term functionality.

Regenerative dentistry, a subset of regenerative medicine, focuses on restoring and regenerating oral tissues. Chitosan's regenerative potential extends to various aspects of regenerative dentistry. For instance, chitosan-based materials could be used in the fabrication of bioactive membranes or drug delivery systems for guided tissue regeneration (GTR) procedures. These membranes can promote the selective growth of specific cell populations while inhibiting the migration of undesired cell types, aiding in the regeneration of damaged oral tissues.

Moreover, chitosan's antimicrobial properties make it a valuable asset in preventing or treating oral infections. Incorporating chitosan into dental materials, such as composites or coatings, may help inhibit bacterial growth, reduce plaque formation, and prevent dental caries.

However, it is important to acknowledge the limitations of pure chitosan scaffolds, such as their poor mechanical properties and rapid degradation rates. These factors can impact their long-term stability and functional outcomes in dental applications. Therefore, combining chitosan with other biomaterials, such as ceramics or polymers, may address these limitations and enhance the performance of chitosan-based constructs in dentistry and regenerative dentistry.

## 7. Conclusions and Future Trends

In the contest of BTE, several studied highlighted the potential use of chitosan as a porous scaffold for BTE because its positive properties, such as biocompatibility, biodegradability and osteoconductive behavior. The translational clinical applications of the use of chitosan could be highly promising in several fields of medicine. Recently, in vivo studies have evaluated the use of chitosan-based matrix for reconstruction of nasal bone defects, demonstrating an improved osteogenesis and a better overall bone density, creating expectation for an improved and time-saving bone healing process 55. Moreover, the new technologies are even more effective in supporting physiological healing processes: as an example, nanomedicine has represented a breakthrough in several tissue engineering procedures. Bone repair is one of the big challenges, where scientists are working to get novel knowledge. Merging novel biomaterials, such as chitosan-based materials, with nanotechnologies based on carbon nanotubes, could be a novel and smart way to face the problem of critical-sized defects 56.

On the other hand, chitosan holds significant potential in dentistry and regenerative dentistry. By leveraging its biocompatibility, biodegradability, and osteoconductive behavior, chitosan-based scaffolds can contribute to periodontal tissue engineering, dental implantology, guided tissue regeneration, and antimicrobial approaches. However, further research, including in vitro and in vivo studies, is essential to fully understand and harness the capabilities of chitosan in these fields. Continued advancements in chitosan-based materials and their application techniques will pave the way for innovative approaches in dentistry, ultimately benefiting patients in need of regenerative dental treatments. Nonetheless, pure chitosan scaffolds have shown poor mechanical properties and rapid degradation rates, which limit their application. Thus, to solve these limitations, it should be focus on combining chitosan with other biomaterials or bioactive molecules to increase their regenerative potential. Chitosan could be considered a potential biomaterial in BTE and clinical dentistry.

## Acknowledge

### Funding

Part of the current activities have been carried out Project **C**ust**O**m**-**made a**NT**ibacterical/bio**A**ctive**/**bio**C**oated prostheses (**CONTACT**) - CUP: B19J20000490005.

### Author Contributions

L.S., G.M., A.F., G.S. and M.S. equally contributed to this paper. B.M., R.M.M., A.V. and L.P. worked on dental applications and critical revision of the current literature. G.M., R.G. and G.S. worked on the revised version of this article. All authors have contributed, in different parts, to this manuscript. All authors have read and agreed to the final revised version of the manuscript.
